# Longitudinal assessment of cortical thickness in healthy older individuals: a comparison between CAT12 and freesurfer

**DOI:** 10.1007/s00234-025-03866-w

**Published:** 2025-12-12

**Authors:** Benita Schmitz-Koep, Vivian Schultz, Fabian Bongratz, Aurore Menegaux, Melissa Thalhammer, Severin Schramm, Su Hwan Kim, Claus Zimmer, Christian Sorg, Christian Wachinger, Panteleimon Giannakopoulos, Marie-Louise Montandon, Cristelle Rodriguez, Sven Haller, Dennis M. Hedderich

**Affiliations:** 1 Department of Neuroradiology, School of Medicine and Health, TUM Klinikum Rechts der Isar, Technical University of Munich, Munich, Germany; 2 TUM-NIC Neuroimaging Center, School of Medicine and Health, Technical University of Munich, Munich, Germany; 3 Laboratory for Artificial Intelligence in Medical Imaging, Department of Radiology, School of Medicine and Health, TUM Klinikum Rechts der Isar, Technical University of Munich, Munich, Germany; 4 Munich Center for Machine Learning, Munich, Germany; 5 Department of Psychiatry, School of Medicine and Health, TUM Klinikum Rechts der Isar, Technical University of Munich, Munich, Germany; 6 Department of Psychiatry, University of Geneva, Geneva, Switzerland; 7 Medical Direction, University of Geneva Hospitals, Geneva, Switzerland; 8 Department of Rehabilitation and Geriatrics, Geneva University Hospitals and University of Geneva, Geneva, Switzerland; 9 CIMC - Centre d’Imagerie Médicale de Cornavin, Geneva, Switzerland; 10 Department of Surgical Sciences, Radiology, Uppsala University, Uppsala, Sweden; 11 Faculty of Medicine, University of Geneva, Geneva, Switzerland; 12 Department of Radiology, Beijing Tiantan Hospital, Capital Medical University, Beijing, China

**Keywords:** CAT12, Cortical thickness, FreeSurfer, Longitudinal, Structural magnetic resonance imaging

## Abstract

**Purpose:**

Cortical thickness (CTh) is a valuable imaging biomarker of brain development and aging. The assessment of CTh using the two most widely utilized software packages, Computational Anatomy Toolbox (CAT12) and FreeSurfer, reveals systematic differences between the two tools. Nonetheless, longitudinal results are supposed to be less affected by such differences. To date, there is no comparison of longitudinal CTh data obtained with these preprocessing pipelines.

**Methods:**

We evaluated cross-sectional and longitudinal measurements of CTh using CAT12 and FreeSurfer in healthy older individuals with structural brain MRI. First, we compared cross-sectional CTh values obtained with these two methods using paired t-tests and correlation analyses. Second, we compared longitudinal CAT12 and FreeSurfer-based data using annualized percent change (APC) of CTh between two timepoints.

**Results:**

Cross-sectional CTh values were higher in FreeSurfer compared to CAT12 in most regions, albeit highly correlated and similarly distributed. In contrast, longitudinal analyses revealed significant differences in APC values with only weak to moderate correlation between the two methods.

**Conclusion:**

Significant differences in longitudinal results between CAT12 and FreeSurfer emphasize the need to consider the preprocessing methods used when interpreting MRI data in brain aging research. Further technical developments are warranted for reliable longitudinal CTh assessment in elderly cohorts.

## Introduction

The mature cerebral neocortex is organized in six horizontal layers and in radial units within which progenitor cells for cortical neurons settle into position after migrating along radial glial fascicles [1]. Structural properties of the cortex can be visualized in vivo using magnetic resonance imaging (MRI). One widely used measure of cortical macrostructure is cortical thickness (CTh), computed as the distance between the pial surface and white matter surface. In healthy adults, a positive relationship between general intelligence and CTh has been found [2, 3], and cortical thinning occurs in the course of healthy aging [4]. Moreover, CTh serves as a biomarker for brain development and aging and exhibits marked changes after preterm birth, in neuropsychiatric disorders, and Alzheimer’s disease [5–9]. The characterization of CTh evolution under normal and pathological conditions has been made possible by large population studies and openly available MRI datasets that imply the increasing importance of automated segmentation. However, preprocessing of structural MRI is not standardized and various tools exist. Two widely used preprocessing software packages are the Computational Anatomy Toolbox (CAT12) and FreeSurfer [10–14]. Both methods were previously assessed in the same databases with conflicting results [15, 16]. However, certain studies postulated that the CTh values obtained with these two methods may be less inconsistent in longitudinal studies [16–19]. Both CAT12, and FreeSurfer offer specific longitudinal pipelines ensuring local comparability across subjects and timepoints by reducing the confounding effect of inter-individual morphological variability as each subject is used as his or her own control [14, 20]. To date, no study has provided a direct comparison of these longitudinal preprocessing pipelines in elderly cohorts. To close this gap, we assessed cross-sectional and longitudinal measurements of CTh in healthy older individuals using structural MRI preprocessed with CAT12 and FreeSurfer.

## Methods

### Participants

The study was performed according to the declaration of Helsinki and approved by the local ethics committee. All participants gave written informed consent prior to inclusion. Subjects were selected from a large population-based longitudinal study on healthy aging that is still ongoing in the Geneva and Lausanne counties and that has been previously described in detail [21–24]: In brief, the cohort included elderly Caucasian white healthy individuals with preserved cognition, living in Geneva and Lausanne catchment area, and recruited via advertisements in local newspapers and media. Due to the need for excellent French knowledge (to participate in detailed neuropsychological testing), most participants were Swiss (or born in French-speaking European countries, 92%). Exclusion criteria included history of psychiatric or neurologic disorders, sustained head injury, history of major medical disorders (neoplasm or cardiac illness), alcohol or drug abuse, regular use of neuroleptics, antidepressants, or psychostimulants, and contraindications to positron emission tomography or MRI. Substantial vascular burden, as evidenced by subtle cardiovascular symptoms, hypertension (non-treated), and a history of stroke or transient ischemic episodes, was an additional exclusion criterion. All individuals included had structural brain MRI at two timepoints (mean period until follow-up: 5 ± 1 years). The final sample included 184 individuals (110 females, mean age at baseline: 73 ± 3 years, mean age at follow-up: 78 ± 3 years).

### MRI data acquisition

At baseline, timepoint 1, imaging data were acquired on a 3 T MRI scanner (TRIO SIEMENS Medical Systems, Erlangen, Germany). The structural high-resolution T1-weighted (T1w) anatomical scan was performed with the following fundamental parameters: 256 × 256 matrix, 176 slices, 1 mm isotropic, TR = 2.27 ms. At follow-up, timepoint 2, high-resolution anatomical 3D T1w data were acquired (254 × 254 matrix, 178 slices, 1 mm isotropic, TR = 7.24 ms) on a 3 T MR750w scanner (GE Healthcare, Milwaukee, Wisconsin). Hence, different scanners at the two timepoints presented a particular challenge for the software packages, which may interact with pipeline behavior and exaggerate differences CTh measurements. We considered applying harmonization techniques to account for scanner differences. However, this was not feasible in our dataset because scanner and timepoint were fully confounded as all baseline scans were acquired on a SIEMENS system and all follow-up scans on a GE system. At both acquisition times, additional sequences (T2-weighted imaging, susceptibility-weighted imaging, diffusion tensor imaging) were used to exclude incidental brain lesions.

### MRI processing

Firstly, 3D T1w data were processed using the longitudinal pipeline CAT12 toolbox (http://www.neuro.uni-jena.de/cat/; version 2137) implemented in SPM12 (version 7771) within Matlab (R2020b) [14]. It includes a fully automated pipeline for surface-based processing to measure CTh using a projection-based thickness method [25]. A workflow specifically tailored towards longitudinal analyses ensures a local comparability both across subjects and across timepoints within subjects and reduces MR-based noise and inhomogeneities, allowing for more sensitive analyses [20, 26, 27]. The pipeline for brain aging (over months, years, and decades) was chosen [14].

Secondly, the longitudinal stream in FreeSurfer (http://surfer.nmr.mgh.harvard.edu/; version 7.1.1) was used to process the T1w images [28]. The surface-based pipeline enables the detection of tissue boundaries, and CTh is calculated as the distance between the white and pial surface [10–13]. The longitudinal stream includes the creation of an unbiased within-subject template space and image [27] using robust, inverse consistent registration [20]. Several processing steps, such as skull stripping, Talairach transforms, atlas registration as well as spherical surface maps and parcellations, are then initialized with common information from the within-subject template, significantly increasing reliability and statistical power [28]. Data were visually inspected for misclassifications during the reconstruction process. No manual editing was performed to evaluate the preprocessing pipelines without introducing subjective bias.

The Desikan-Killiany Atlas was used to subdivide the surface into 68 gyral-based regions of interest (ROIs), 34 per hemisphere, and CTh values were extracted within these ROIs for both CAT12 and FreeSurfer using the respective standard procedures [29]. Values with a z-score greater than + 3 or less than − 3 were considered as outliers and removed.

### Statistical analysis

All statistical analyses were performed using IBM SPSS Version 28 (IBM Corp., Armonk, NY, USA) and corrected for multiple comparisons to control the false discovery rate (FDR) using the Benjamini–Hochberg procedure [30]. Statistical significance was defined as *p* < 0.05, FDR-corrected.

#### Cross-sectional comparison at timepoint 1 and 2

First, for visual inspection, the mean and standard deviation (SD) of CTh per ROI across all individuals were plotted onto the brain surface using Simple Brain Plot [31] for CAT12 and for FreeSurfer, for timepoint 1 and timepoint 2, respectively.

To test whether CTh values are significantly different between the two methods, paired t-tests between CAT12 and FreeSurfer for both timepoint 1 and timepoint 2 were performed separately. Results were FDR-corrected for multiple comparisons across all 68 ROIs.

Furthermore, we analyzed the correlation between CTh values in CAT12 and values in FreeSurfer per ROI for both timepoint 1 and timepoint 2 separately. Results were FDR-corrected for multiple comparisons across all 68 ROIs.

#### Longitudinal comparison

To evaluate longitudinal differences between measurements of CTh, annualized percent change (APC) was calculated for each ROI using the following equation:

$$\:\frac{{CTh}_{timepoint\:2}\:-{CTh}_{timepoint\:1}}{{CTh}_{timepoint\:1}}*\frac{1}{time\:interval}*100$$

The time interval was the time between timepoint 1 and timepoint 2 in years.

First, for visual inspection, mean and SD of APC per ROI across all individuals were plotted onto the brain surface using Simple Brain Plot [31] for CAT12 and for FreeSurfer.

To test whether APC values are significantly different between the two methods, paired t-tests were performed for each ROI between CAT12 and FreeSurfer. Results were FDR-corrected for multiple comparisons across all 68 ROIs.

Furthermore, we analyzed the correlation between APC in CAT12 and APC in FreeSurfer per ROI. Results were FDR-corrected for multiple comparisons across all 68 ROIs.

### Data Availability

Participant data used in this study are not publicly available but stored by the principal investigators of the Geneva Aging Study.

## Results

### Effects of preprocessing on cross-sectional results

**Fig. 1:**
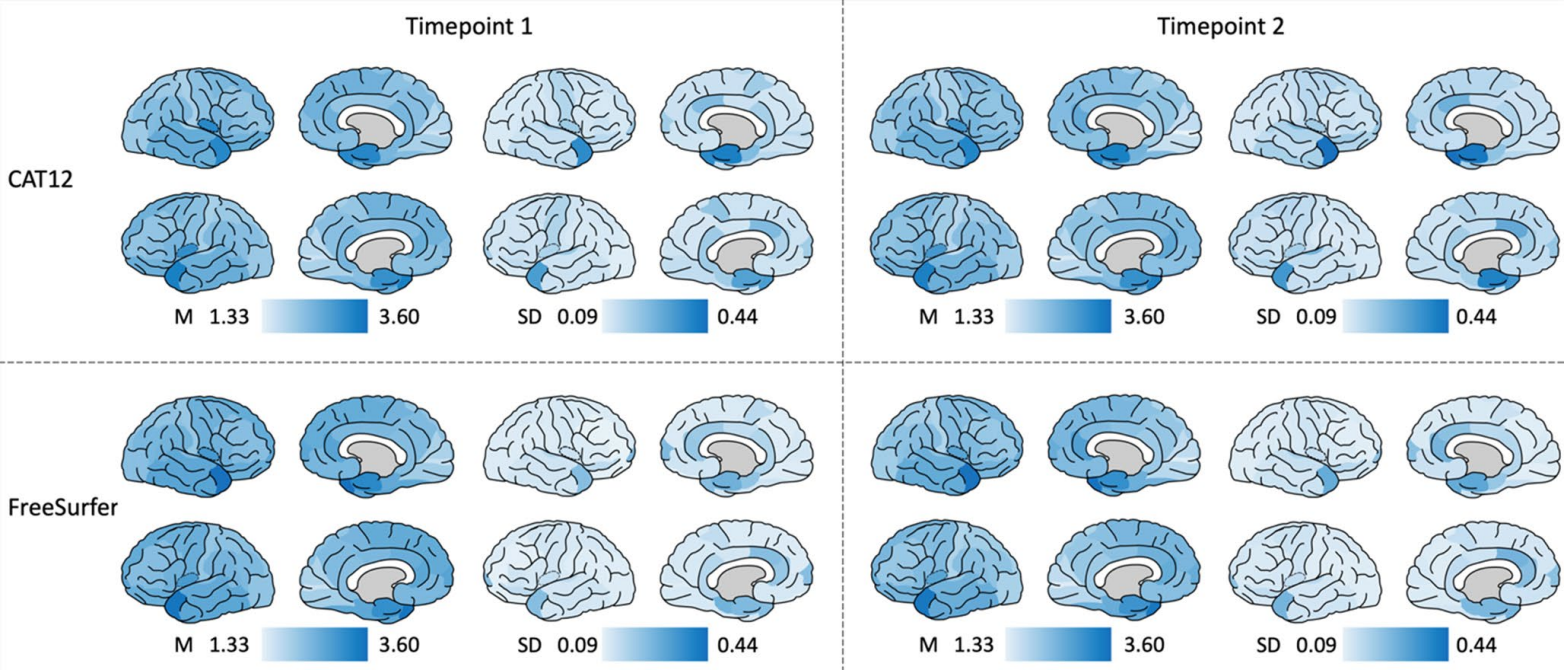
Cortical thickness at timepoint 1 and timepoint 2. Surface maps of mean and SD of CTh (in mm) at timepoint 1 and timepoint 2 in CAT12 and FreeSurfer. Values are color-coded, darker colors indicate higher values. Both hemispheres are shown in medial and lateral views. Abbreviations: *M* mean, *SD* standard deviation

Figure 1 shows the mean and SD of CTh (in mm) per ROI across all individuals plotted onto the brain surface for CAT12 and for FreeSurfer, for timepoint 1 and timepoint 2 separately. On visual inspection, cross-sectional CTh was similarly distributed in CAT12 and FreeSurfer. However, CTh values in FreeSurfer were higher compared to values in CAT12 at both timepoints. Both CAT12 and FreeSurfer showed higher SD in the medial temporal lobes and at the temporal poles. However, SD of CTh values in CAT12 were significantly higher compared to FreeSurfer.

In most ROIs, CTh values in FreeSurfer were significantly higher compared to values in CAT12 at both timepoints. There were, however, some exceptions. CTh values in left and right insula were significantly higher in CAT12 compared to FreeSurfer at both timepoints. This was also the case for CTh value in right entorhinal cortex at timepoint 2. Furthermore, there was no significant difference between the two methods for CTh values in left and right banks of the superior temporal sulcus, left and right entorhinal cortex, left lateral orbitofrontal cortex, and left and right pericalcarine cortex at timepoint 1, and left entorhinal and left posterior cingulate cortex at timepoint 2. Table 1 lists t- and p-values per ROI for timepoint 1 and for timepoint 2.

**Table 1:** Cross-sectional results of paired t-test

ROI	Timepoint 1		Timepoint 2	
	t-value	p-value	t-value	p-value
Left hemisphere				
Banks of the superior temporal sulcus	1.062	0.29	-7.284	**<0.001**
Caudal anterior cingulate cortex	-16.792	**<0.001**	-13.345	**<0.001**
Caudal middle frontal gyrus	-27.091	**<0.001**	-17.719	**<0.001**
Cuneus cortex	-14.929	**<0.001**	-19.42	**<0.001**
Entorhinal cortex	-0.496	0.62	0.198	0.843
Fusiform gyrus	-33.104	**<0.001**	-27.328	**<0.001**
Inferior parietal cortex	-17.155	**<0.001**	-16.757	**<0.001**
Inferior temporal gyrus	-13.957	**<0.001**	-28.428	**<0.001**
Isthmus of the cingulate cortex	10.538	**<0.001**	-8.837	**<0.001**
Lateral occipital cortex	-13.016	**<0.001**	-27.497	**<0.001**
Lateral orbitofrontal cortex	1.863	0.064	-11.087	**<0.001**
Lingual gyrus	-7.547	**<0.001**	-30.537	**<0.001**
Medial orbitofrontal cortex	-18.508	**<0.001**	-20.08	**<0.001**
Middle temporal gyrus	-12.752	**<0.001**	-22.241	**<0.001**
Parahippocampal gyrus	-26.605	**<0.001**	-29.864	**<0.001**
Paracentral lobule	-23.825	**<0.001**	-37.111	**<0.001**
Inferior frontal gyrus, pars opercularis	-10.917	**<0.001**	-14.59	**<0.001**
Inferior frontal gyrus, pars orbitalis	-13.556	**<0.001**	-21.692	**<0.001**
Inferior frontal gyrus, pars triangularis	-4.93	**<0.001**	-12.762	**<0.001**
Pericalcarine cortex	-1.38	0.169	-21.257	**<0.001**
Postcentral gyrus	-32.834	**<0.001**	-42.109	**<0.001**
Posterior cingulate cortex	-14.328	**<0.001**	-0.18	0.858
Precentral gyrus	-34.413	**<0.001**	-54.562	**<0.001**
Precuneus cortex	-14.133	**<0.001**	-15.419	**<0.001**
Rostral anterior cingulate cortex	-10.914	**<0.001**	-4.629	**<0.001**
Rostral middle frontal gyrus	-22.723	**<0.001**	-18.277	**<0.001**
Superior frontal gyrus	-24.265	**<0.001**	-26.695	**<0.001**
Superior parietal cortex	-38.598	**<0.001**	-35.238	**<0.001**
Superior temporal gyrus	-15.439	**<0.001**	-29.851	**<0.001**
Supramarginal gyrus	-22.786	**<0.001**	-28.651	**<0.001**
Frontal pole	-27.177	**<0.001**	-25.548	**<0.001**
Temporal pole	-8.667	**<0.001**	-9.11	**<0.001**
Transverse temporal gyrus	-24.332	**<0.001**	-36.512	**<0.001**
Insula	22.783	**<0.001**	10.889	**<0.001**
Right hemisphere				
Banks of the superior temporal sulcus	-0.196	0.845	-11.928	**<0.001**
Caudal anterior cingulate cortex	-11.772	**<0.001**	-11.236	**<0.001**
Caudal middle frontal gyrus	-19.276	**<0.001**	-15.467	**<0.001**
Cuneus cortex	-16.173	**<0.001**	-27.44	**<0.001**
Entorhinal cortex	1.364	0.174	6.841	**<0.001**
Fusiform gyrus	-38.963	**<0.001**	-30.475	**<0.001**
Inferior parietal cortex	-20.715	**<0.001**	-14.515	**<0.001**
Inferior temporal gyrus	-18.243	**<0.001**	-33.795	**<0.001**
Isthmus of the cingulate cortex	8.018	**<0.001**	-15.187	**<0.001**
Lateral occipital cortex	-30.823	**<0.001**	-31.86	**<0.001**
Lateral orbitofrontal cortex	9.758	**<0.001**	-21.423	**<0.001**
Lingual gyrus	-9.932	**<0.001**	-43.853	**<0.001**
Medial orbitofrontal cortex	-12.937	**<0.001**	-24.562	**<0.001**
Middle temporal gyrus	-10.758	**<0.001**	-16.455	**<0.001**
Parahippocampal gyrus	-14.39	**<0.001**	-15.8	**<0.001**
Paracentral lobule	-23.328	**<0.001**	-40.361	**<0.001**
Inferior frontal gyrus, pars opercularis	-7.528	**<0.001**	-12.117	**<0.001**
Inferior frontal gyrus, pars orbitalis	-12.273	**<0.001**	-20.126	**<0.001**
Inferior frontal gyrus, pars triangularis	-4.246	**<0.001**	-12.09	**<0.001**
Pericalcarine cortex	-1.239	0.217	-36.401	**<0.001**
Postcentral gyrus	-24.911	**<0.001**	-39.295	**<0.001**
Posterior cingulate cortex	-19.183	**<0.001**	-4.065	**<0.001**
Precentral gyrus	-31.299	**<0.001**	-53.101	**<0.001**
Precuneus cortex	-12.559	**<0.001**	-18.977	**<0.001**
Rostral anterior cingulate cortex	-12.943	**<0.001**	-14.097	**<0.001**
Rostral middle frontal gyrus	-20.793	**<0.001**	-16.483	**<0.001**
Superior frontal gyrus	-19.948	**<0.001**	-24.989	**<0.001**
Superior parietal cortex	-29.618	**<0.001**	-30.629	**<0.001**
Superior temporal gyrus	-9.212	**<0.001**	-26.378	**<0.001**
Supramarginal gyrus	-22.964	**<0.001**	-27.187	**<0.001**
Frontal pole	-29.129	**<0.001**	-27.604	**<0.001**
Temporal pole	-14	**<0.001**	-11.03	**<0.001**
Transverse temporal gyrus	-25.604	**<0.001**	-24.783	**<0.001**
Insula	25.913	**<0.001**	16.469	**<0.001**

T-values and p-values of the paired t-tests between CTh values in CAT12 and FreeSurfer for timepoint 1 and timepoint 2

Bold letters indicate statistical significance defined as p<0.05, FDR-corrected

Abbreviations: *ROI* region of interest

**Fig. 2:**
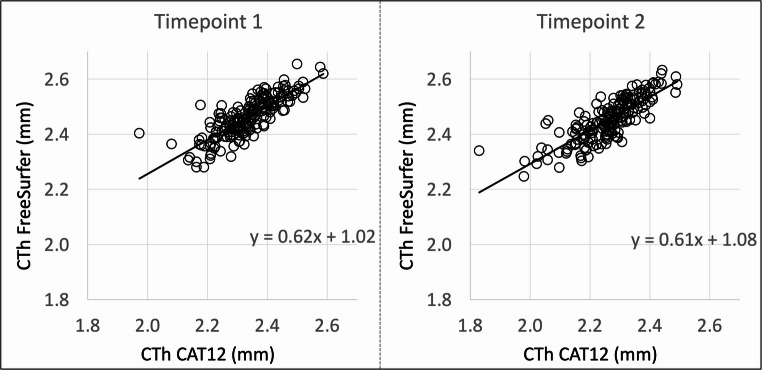
Relationship between cortical thickness values in CAT12 and FreeSurfer at timepoint 1 and timepoint 2. Scatter plots of CTh in CAT12 and CTh in FreeSurfer (in mm) across all regions at timepoint 1 and timepoint 2. CTh in CAT12 in millimeters is plotted on the x-axes; CTh in FreeSurfer in millimeters is plotted on the y-axes. Linear regression lines were added. Abbreviation: *CTh* cortical thickness

Moreover, we analyzed the correlation between CTh values in CAT12 and FreeSurfer. Except for left and right entorhinal cortex at timepoint 1, values of CAT12 and FreeSurfer correlated significantly in all ROIs at both timepoints. In most of the ROIs, the correlation was strong (*r*≥0.600) at both timepoints, while some regions displayed moderate (*r* = 0.400–0.590) and only few regions weak correlations (*r* < 0.400). Table 2 lists correlation coefficients and p-values per ROI for timepoint 1 and for timepoint 2. Figure 2 shows a scatter plot of CTh across all 68 ROIs in CAT12 and FreeSurfer at timepoint 1 and at timepoint 2

**Table 2:** Cross-sectional results of correlation analysis

ROI	Timepoint 1		Timepoint 2	
	r	p-value	r	p-value
Left hemisphere				
Banks of the superior temporal sulcus	0.799	**<0.001**	0.764	**<0.001**
Caudal anterior cingulate cortex	0.625	**<0.001**	0.649	**<0.001**
Caudal middle frontal gyrus	0.846	**<0.001**	0.804	**<0.001**
Cuneus cortex	0.698	**<0.001**	0.616	**<0.001**
Entorhinal cortex	0.146	0.05	0.42	**<0.001**
Fusiform gyrus	0.587	**<0.001**	0.668	**<0.001**
Inferior parietal cortex	0.8	**<0.001**	0.86	**<0.001**
Inferior temporal gyrus	0.55	**<0.001**	0.682	**<0.001**
Isthmus of the cingulate cortex	0.443	**<0.001**	0.406	**<0.001**
Lateral occipital cortex	0.678	**<0.001**	0.731	**<0.001**
Lateral orbitofrontal cortex	0.574	**<0.001**	0.649	**<0.001**
Lingual gyrus	0.651	**<0.001**	0.606	**<0.001**
Medial orbitofrontal cortex	0.551	**<0.001**	0.543	**<0.001**
Middle temporal gyrus	0.717	**<0.001**	0.785	**<0.001**
Parahippocampal gyrus	0.609	**<0.001**	0.571	**<0.001**
Paracentral lobule	0.651	**<0.001**	0.722	**<0.001**
Inferior frontal gyrus, pars opercularis	0.808	**<0.001**	0.802	**<0.001**
Inferior frontal gyrus, pars orbitalis	0.636	**<0.001**	0.646	**<0.001**
Inferior frontal gyrus, pars triangularis	0.794	**<0.001**	0.814	**<0.001**
Pericalcarine cortex	0.422	**<0.001**	0.508	**<0.001**
Postcentral gyrus	0.797	**<0.001**	0.781	**<0.001**
Posterior cingulate cortex	0.337	**<0.001**	0.302	**<0.001**
Precentral gyrus	0.737	**<0.001**	0.694	**<0.001**
Precuneus cortex	0.81	**<0.001**	0.796	**<0.001**
Rostral anterior cingulate cortex	0.577	**<0.001**	0.594	**<0.001**
Rostral middle frontal gyrus	0.77	**<0.001**	0.864	**<0.001**
Superior frontal gyrus	0.85	**<0.001**	0.715	**<0.001**
Superior parietal cortex	0.92	**<0.001**	0.919	**<0.001**
Superior temporal gyrus	0.773	**<0.001**	0.777	**<0.001**
Supramarginal gyrus	0.817	**<0.001**	0.845	**<0.001**
Frontal pole	0.538	**<0.001**	0.627	**<0.001**
Temporal pole	0.434	**<0.001**	0.588	**<0.001**
Transverse temporal gyrus	0.643	**<0.001**	0.535	**<0.001**
Insula	0.702	**<0.001**	0.74	**<0.001**
Right hemisphere				
Banks of the superior temporal sulcus	0.798	**<0.001**	0.747	**<0.001**
Caudal anterior cingulate cortex	0.248	**0.001**	0.365	**<0.001**
Caudal middle frontal gyrus	0.764	**<0.001**	0.749	**<0.001**
Cuneus cortex	0.705	**<0.001**	0.577	**<0.001**
Entorhinal cortex	0.13	0.081	0.476	**<0.001**
Fusiform gyrus	0.584	**<0.001**	0.681	**<0.001**
Inferior parietal cortex	0.799	**<0.001**	0.861	**<0.001**
Inferior temporal gyrus	0.576	**<0.001**	0.66	**<0.001**
Isthmus of the cingulate cortex	0.495	**<0.001**	0.374	**<0.001**
Lateral occipital cortex	0.696	**<0.001**	0.625	**<0.001**
Lateral orbitofrontal cortex	0.534	**<0.001**	0.6	**<0.001**
Lingual gyrus	0.649	**<0.001**	0.581	**<0.001**
Medial orbitofrontal cortex	0.399	**<0.001**	0.509	**<0.001**
Middle temporal gyrus	0.732	**<0.001**	0.742	**<0.001**
Parahippocampal gyrus	0.63	**<0.001**	0.734	**<0.001**
Paracentral lobule	0.677	**<0.001**	0.632	**<0.001**
Inferior frontal gyrus, pars opercularis	0.786	**<0.001**	0.808	**<0.001**
Inferior frontal gyrus, pars orbitalis	0.678	**<0.001**	0.71	**<0.001**
Inferior frontal gyrus, pars triangularis	0.761	**<0.001**	0.781	**<0.001**
Pericalcarine cortex	0.49	**<0.001**	0.508	**<0.001**
Postcentral gyrus	0.752	**<0.001**	0.712	**<0.001**
Posterior cingulate cortex	0.368	**<0.001**	0.309	**0.006**
Precentral gyrus	0.717	**<0.001**	0.648	**<0.001**
Precuneus cortex	0.834	**<0.001**	0.738	**<0.001**
Rostral anterior cingulate cortex	0.278	**<0.001**	0.415	**<0.001**
Rostral middle frontal gyrus	0.73	**<0.001**	0.81	**<0.001**
Superior frontal gyrus	0.834	**<0.001**	0.683	**<0.001**
Superior parietal cortex	0.875	**<0.001**	0.903	**<0.001**
Superior temporal gyrus	0.797	**<0.001**	0.758	**<0.001**
Supramarginal gyrus	0.833	**<0.001**	0.868	**<0.001**
Frontal pole	0.62	**<0.001**	0.632	**<0.001**
Temporal pole	0.316	**<0.001**	0.495	**<0.001**
Transverse temporal gyrus	0.465	**<0.001**	0.437	**<0.001**
Insula	0.622	**<0.001**	0.7	**<0.001**

Correlation coefficients and p-values of the correlation analyses between CTh values in CAT12 and FreeSurfer for timepoint 1 and timepoint 2

Bold letters indicate statistical significance defined as p<0.05, FDR-corrected

Abbreviations: *ROI* region of interest

### Effects of preprocessing on longitudinal results

**Fig. 3:**
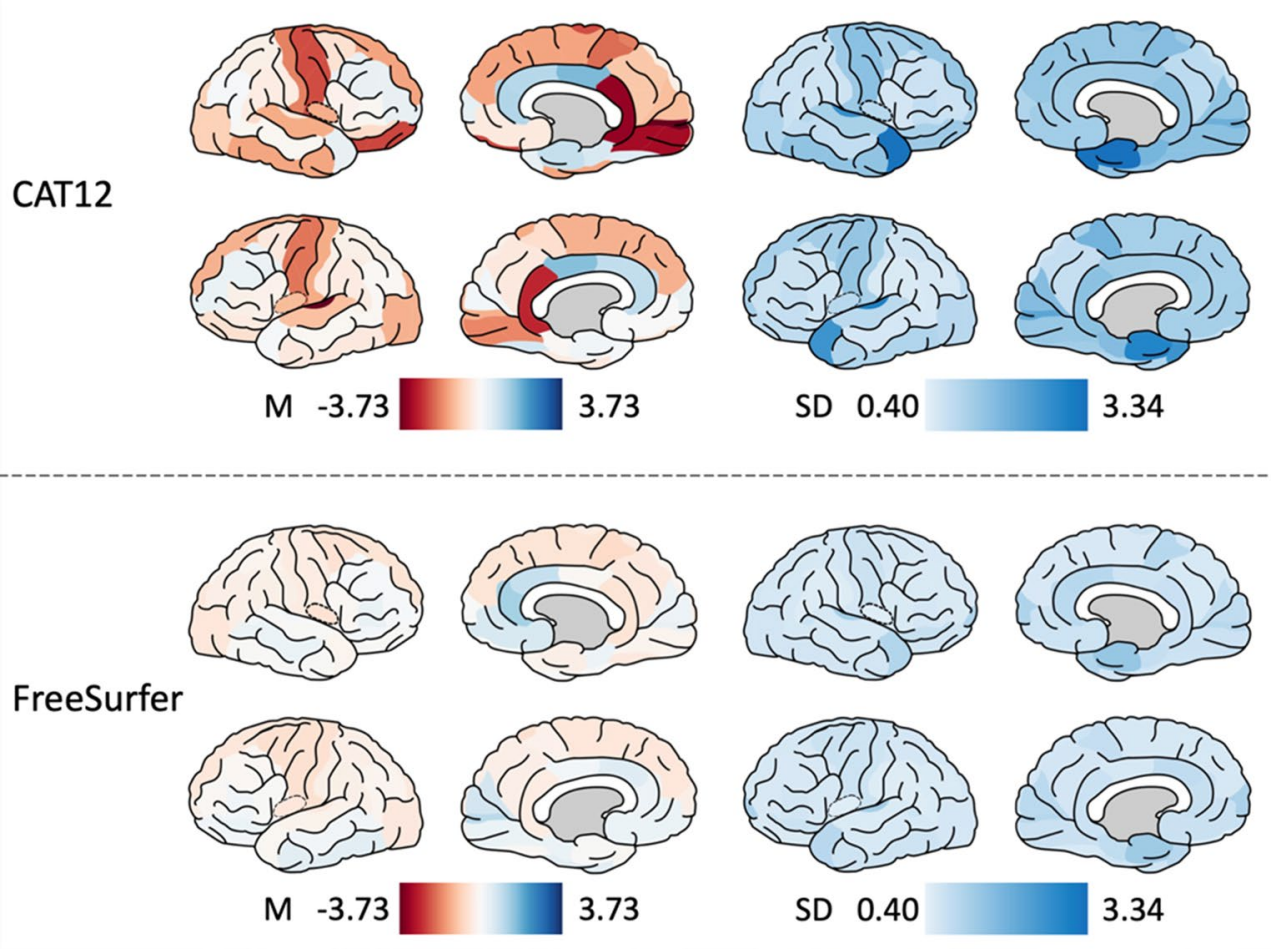
Annualized percent change of cortical thickness. Surface maps of mean and SD of APC (in %) in CAT12 and FreeSurfer. Values are color-coded, red colors indicate negative APC, blue colors indicate positive APC. Both hemispheres are shown in medial and lateral views. Abbreviations: *M* mean, *SD* standard deviation

Figure 3 shows the mean and SD of APC (in %) per ROI across all individuals plotted onto the brain surface for CAT12 and FreeSurfer. On visual inspection, APC in CAT12 was much higher in CAT12 compared to FreeSurfer, particularly in left transverse temporal gyrus, left and right isthmus of the cingulate cortex, left and right precentral gyrus, right lateral orbitofrontal cortex, right lingual gyrus, and right paracentral lobule. In these areas, the APC range in CAT12 was between -2 to -3%. Similar to the cross-sectional results, both CAT12 and FreeSurfer showed higher SD in the medial temporal lobes and temporal poles. The APC SD values in CAT12 were significantly higher compared to FreeSurfer.

In most ROIs, APC was negative and significantly lower in CAT12 compared to FreeSurfer, which means that in most ROIs, values in CAT12 indicated more severe thinning of the cortex between timepoint 1 and timepoint 2. However, APC was positive and significantly higher in CAT12 compared to FreeSurfer in left and right fusiform gyrus, left and right posterior cingulate cortex, left and right rostral middle frontal gyrus, left and right frontal pole, left caudal anterior cingulate cortex, left rostral anterior cingulate cortex, right entorhinal cortex, right inferior parietal cortex, right parahippocampal gyrus, and right temporal pole. There was no significant difference between the two methods for APC in left and right superior parietal cortex, left entorhinal cortex, left temporal pole, right caudal anterior cingulate cortex, right caudal middle frontal gyrus, right rostral anterior cingulate cortex, and right supramarginal gyrus. Table 3 lists APC in CAT12 and FreeSurfer, as well as t- and p-values per ROI.

**Table 3:** Longitudinal results of paired t-test

ROI	APC values		Paired t-test	
	CAT12	FreeSurfer	t-value	p-value
Left hemisphere				
Banks of the superior temporal sulcus	-0.22	0.255	-7.991	**<0.001**
Caudal anterior cingulate cortex	0.952	0.498	4.835	**<0.001**
Caudal middle frontal gyrus	-0.465	-0.618	2.143	**0.033**
Cuneus cortex	0.142	0.721	-4.598	**<0.001**
Entorhinal cortex	0.159	-0.037	0.958	0.339
Fusiform gyrus	0.681	-0.077	8.354	**<0.001**
Inferior parietal cortex	-0.066	-0.183	2.396	**0.018**
Inferior temporal gyrus	-0.435	0.455	-10.366	**<0.001**
Isthmus of the cingulate cortex	-2.794	-0.36	-20.999	**<0.001**
Lateral occipital cortex	-1.341	-0.47	-11.828	**<0.001**
Lateral orbitofrontal cortex	-0.632	0.167	-12.88	**<0.001**
Lingual gyrus	-1.834	0.277	-20.751	**<0.001**
Medial orbitofrontal cortex	0.018	0.275	-3.15	**0.002**
Middle temporal gyrus	-0.14	0.21	-6.541	**<0.001**
Parahippocampal gyrus	-0.06	0.244	-2.641	**0.009**
Paracentral lobule	-0.651	-0.236	-3.187	**0.002**
Inferior frontal gyrus, pars opercularis	-0.24	-0.051	-3.908	**<0.001**
Inferior frontal gyrus, pars orbitalis	-0.399	0.275	-9.56	**<0.001**
Inferior frontal gyrus, pars triangularis	-0.269	0.171	-7.454	**<0.001**
Pericalcarine cortex	-1.352	1.05	-15.663	**<0.001**
Postcentral gyrus	-1.403	-0.594	-11.506	**<0.001**
Posterior cingulate cortex	1.606	0.147	15.648	**<0.001**
Precentral gyrus	-1.994	-0.602	-15.624	**<0.001**
Precuneus cortex	-0.463	-0.325	-2.145	**0.033**
Rostral anterior cingulate cortex	1.002	0.257	8.61	**<0.001**
Rostral middle frontal gyrus	0.297	-0.081	8.066	**<0.001**
Superior frontal gyrus	-1.315	-0.466	-11.499	**<0.001**
Superior parietal cortex	-0.274	-0.295	0.495	0.621
Superior temporal gyrus	-1.158	-0.227	-13.958	**<0.001**
Supramarginal gyrus	-0.39	-0.263	-2.985	**0.003**
Frontal pole	0.304	-0.296	5.477	**<0.001**
Temporal pole	0.047	-0.106	0.863	0.389
Transverse temporal gyrus	-3.732	-0.473	-15.809	**<0.001**
Insula	-1.216	-0.467	-9.976	**<0.001**
Right hemisphere				
Banks of the superior temporal sulcus	-0.723	0.132	-9.305	**<0.001**
Caudal anterior cingulate cortex	1.07	0.883	1.944	0.053
Caudal middle frontal gyrus	-0.598	-0.572	-0.35	0.727
Cuneus cortex	-1.195	0.251	-12.476	**<0.001**
Entorhinal cortex	0.898	-0.274	4.924	**<0.001**
Fusiform gyrus	0.519	-0.45	8.983	**<0.001**
Inferior parietal cortex	0.071	-0.305	6.64	**<0.001**
Inferior temporal gyrus	-1.461	-0.028	-14.163	**<0.001**
Isthmus of the cingulate cortex	-3.318	-0.529	-26.057	**<0.001**
Lateral occipital cortex	-0.913	-0.503	-4.937	**<0.001**
Lateral orbitofrontal cortex	-2.48	-0.049	-27.454	**<0.001**
Lingual gyrus	-3.138	-0.191	-27.652	**<0.001**
Medial orbitofrontal cortex	-0.421	0.624	-10.784	**<0.001**
Middle temporal gyrus	-0.121	0.288	-5.924	**<0.001**
Parahippocampal gyrus	0.623	0.258	2.88	**0.004**
Paracentral lobule	-2.092	-0.719	-11.899	**<0.001**
Inferior frontal gyrus, pars opercularis	-0.256	-0.054	-4.149	**<0.001**
Inferior frontal gyrus, pars orbitalis	-0.687	0.124	-9.797	**<0.001**
Inferior frontal gyrus, pars triangularis	-0.135	0.322	-6.874	**<0.001**
Pericalcarine cortex	-3.715	0.344	-27.795	**<0.001**
Postcentral gyrus	-1.876	-0.383	-17.252	**<0.001**
Posterior cingulate cortex	1.676	-0.058	17.964	**<0.001**
Precentral gyrus	-2.407	-0.36	-21.532	**<0.001**
Precuneus cortex	-1.057	-0.464	-8.42	**<0.001**
Rostral anterior cingulate cortex	1.129	1.288	-1.597	0.112
Rostral middle frontal gyrus	0.339	0.039	6.443	**<0.001**
Superior frontal gyrus	-1.492	-0.542	-10.755	**<0.001**
Superior parietal cortex	-0.264	-0.178	-1.69	0.093
Superior temporal gyrus	-1.415	-0.048	-16.012	**<0.001**
Supramarginal gyrus	-0.347	-0.276	-1.338	0.183
Frontal pole	0.39	0.001	3.876	**<0.001**
Temporal pole	0.369	-0.194	2.539	**0.012**
Transverse temporal gyrus	-0.479	0.149	-3.062	**0.003**
Insula	-1.364	-0.572	-10.676	**<0.001**

T-values and p-values of the paired t-tests between CTh values in CAT12 and FreeSurfer for timepoint 1 and timepoint 2

Bold letters indicate statistical significance defined as p<0.05, FDR-corrected

Abbreviations: *ROI* region of interest

**Fig. 4:**
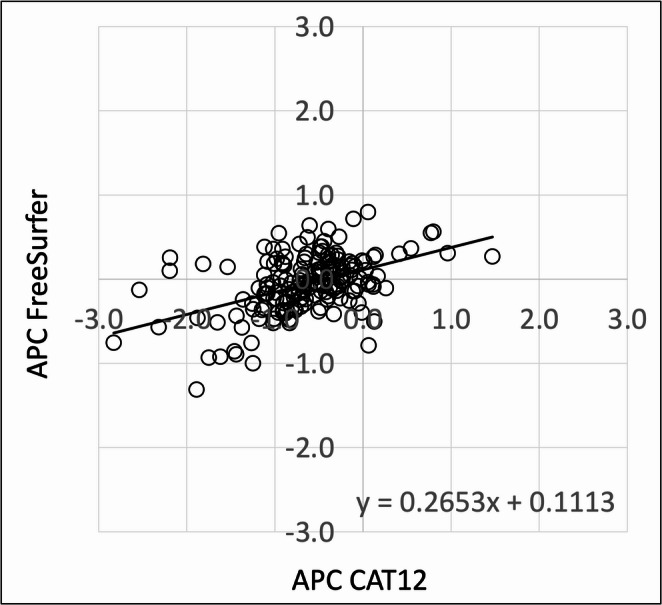
Relationship between annualized percent change in CAT12 and FreeSurfer. Scatter plot of APC in CAT12 and APC in FreeSurfer (in %) across all regions. APC in CAT12 is plotted on the x-axis; APC in FreeSurfer is plotted on the y-axis. A linear regression line was added. Abbreviation: *APC* annualized percent change

Except for the right banks of the superior temporal sulcus, APC in CAT12 and FreeSurfer correlated significantly in all ROIs. In most of the ROIs, the correlation was weak. A few regions displayed moderate correlations between the two methods. Table 4 lists correlation coefficients and p-values per ROI. Figure 4 shows a scatter plot of APC across all 68 ROIs in CAT12 and FreeSurfer

**Table 4:** Longitudinal results of correlation analysis

**ROI**	**r**	**p-value**
Left hemisphere		
Banks of the superior temporal sulcus	0.293	**<0.001**
Caudal anterior cingulate cortex	0.34	**<0.001**
Caudal middle frontal gyrus	0.621	**<0.001**
Cuneus cortex	0.224	**<0.001**
Entorhinal cortex	0.313	**<0.001**
Fusiform gyrus	0.319	**<0.001**
Inferior parietal cortex	0.353	**<0.001**
Inferior temporal gyrus	0.187	**0.012**
Isthmus of the cingulate cortex	0.192	**0.01**
Lateral occipital cortex	0.291	**<0.001**
Lateral orbitofrontal cortex	0.413	**<0.001**
Lingual gyrus	0.285	**<0.001**
Medial orbitofrontal cortex	0.453	**<0.001**
Middle temporal gyrus	0.292	**<0.001**
Parahippocampal gyrus	0.362	**<0.001**
Paracentral lobule	0.323	**<0.001**
Inferior frontal gyrus, pars opercularis	0.493	**<0.001**
Inferior frontal gyrus, pars orbitalis	0.283	**<0.001**
Inferior frontal gyrus, pars triangularis	0.416	**<0.001**
Pericalcarine cortex	0.329	**<0.001**
Postcentral gyrus	0.576	**<0.001**
Posterior cingulate cortex	0.3	**<0.001**
Precentral gyrus	0.568	**<0.001**
Precuneus cortex	0.285	**<0.001**
Rostral anterior cingulate cortex	0.342	**<0.001**
Rostral middle frontal gyrus	0.568	**<0.001**
Superior frontal gyrus	0.537	**<0.001**
Superior parietal cortex	0.627	**<0.001**
Superior temporal gyrus	0.374	**<0.001**
Supramarginal gyrus	0.404	**<0.001**
Frontal pole	0.22	**0.003**
Temporal pole	0.308	**<0.001**
Transverse temporal gyrus	0.381	**0.004**
Insula	0.213	**<0.001**
Right hemisphere		
Banks of the superior temporal sulcus	-0.012	0.867
Caudal anterior cingulate cortex	0.351	**<0.001**
Caudal middle frontal gyrus	0.529	**<0.001**
Cuneus cortex	0.217	**0.003**
Entorhinal cortex	0.302	**<0.001**
Fusiform gyrus	0.354	**<0.001**
Inferior parietal cortex	0.361	**<0.001**
Inferior temporal gyrus	0.367	**<0.001**
Isthmus of the cingulate cortex	0.291	**<0.001**
Lateral occipital cortex	0.233	**<0.001**
Lateral orbitofrontal cortex	0.458	**<0.001**
Lingual gyrus	0.386	**<0.001**
Medial orbitofrontal cortex	0.205	**0.006**
Middle temporal gyrus	0.239	**0.001**
Parahippocampal gyrus	0.332	**<0.001**
Paracentral lobule	0.366	**<0.001**
Inferior frontal gyrus, pars opercularis	0.406	**<0.001**
Inferior frontal gyrus, pars orbitalis	0.37	**<0.001**
Inferior frontal gyrus, pars triangularis	0.255	**0.001**
Pericalcarine cortex	0.217	**0.003**
Postcentral gyrus	0.469	**<0.001**
Posterior cingulate cortex	0.286	**<0.001**
Precentral gyrus	0.563	**<0.001**
Precuneus cortex	0.265	**<0.001**
Rostral anterior cingulate cortex	0.284	**<0.001**
Rostral middle frontal gyrus	0.553	**<0.001**
Superior frontal gyrus	0.528	**<0.001**
Superior parietal cortex	0.483	**<0.001**
Superior temporal gyrus	0.282	**<0.001**
Supramarginal gyrus	0.318	**<0.001**
Frontal pole	0.246	**<0.001**
Temporal pole	0.425	**<0.001**
Transverse temporal gyrus	0.154	**0.038**
Insula	0.192	**0.01**

Correlation coefficients and p-values of the correlation analyses between APC in CAT12 and FreeSurfer

Bold letters indicate statistical significance defined as p<0.05, FDR-corrected

Abbreviations: *ROI* region of interest

## Discussion

In the present study, we compared cross-sectional and longitudinal results of CAT12 and FreeSurfer CTh measurements using structural MRI, facing a particular challenge due to using different scanners at the two timepoints. While regional distribution of cross-sectional CTh was similar between the two methods with high correlation coefficients, our longitudinal analysis led to radically different conclusions.

### Effects of preprocessing on cross-sectional results

In most ROIs, CTh values in FreeSurfer were significantly higher than values in CAT12 at both timepoints. However, values of CAT12 and FreeSurfer were highly correlated in most ROIs.

In line with our results, several studies reported high correlation between measures of CAT12 and FreeSurfer [15, 16, 18, 19, 32]. Furthermore, Righart et al. [15] found systematically higher CTh values using FreeSurfer compared to CAT12. Some studies did not confirm this point of view: Lower values in FreeSurfer compared to CAT 12 have been also reported [16, 32].

###  Effects of preprocessing on longitudinal results

Previous studies reported excellent test-retest reliability for both CAT12 and FreeSurfer, and therefore concluded that longitudinal results should be less affected by systematic differences due to preprocessing [16–19].

The present study showed a significant difference between APC in CAT12 compared to FreeSurfer in most ROIs. This difference corresponded frequently but not consistently to more severe thinning of the cortex obtained with CAT12 compared to FreeSurfer. APC values of CAT12 and FreeSurfer showed weak to moderate correlations.

Our results indicate that the choice of the preprocessing methods may significantly affect the longitudinal CTh results, at least in case of different scanner platforms between two timepoints. In those regions that showed the most severe thinning between the two timepoints, such as left transverse temporal gyrus, left and right isthmus of the cingulate cortex, left and right precentral gyrus, right lateral orbitofrontal cortex, right lingual gyrus, and right paracentral lobule, APC in CAT12 was about − 2 to -3%, whereas APC in FreeSurfer was about − 0.4%. Previous studies using alternative preprocessing methods (SIENA and SIENAX software as part of the FMRIB Software Library, www.fmrib.ox.ac.uk/fsl; and a different validated, open-source segmentation tool [33, 34]) reported APC values for whole brain atrophy between − 0.4% and − 0.45% [35, 36]. However, it remains unclear which pipeline more accurately reflects true cortical changes, as no external ground truth is available for direct validation. This uncertainty highlights the importance of carefully considering preprocessing methods when interpreting longitudinal CTh results and the need for further research on validation approaches, such as manual segmentation [37, 38], histological comparisons [39, 40], and phantom studies [25, 41].

Scanner differences may also have played a critical role in the observed discrepancies between pipelines. At baseline (timepoint 1), a 3 T SIEMENS scanner was used, while follow-up scans (timepoint 2) were acquired on a 3 T GE Healthcare system. Although both software packages processed images from the same scanners, the confounding effect of scanner change may interact differently with each preprocessing pipeline, potentially exaggerating differences in longitudinal CTh estimates. Our results suggest that FreeSurfer may be less sensitive to scanner-induced variability than CAT12, though without external validation we cannot definitively conclude which tool is more robust. Future longitudinal studies should prioritize scanner consistency or incorporate scanner harmonization methods when applicable.

One limitation of the current study is the use of different scanners at timepoint 1 and timepoint 2. At timepoint 1, a 3 T SIEMENS scanner was used, at timepoint 2, a 3 T GE Healthcare. While this difference was the same for both software packages, the magnitude of the confounding effect could differ between software packages. Another important limitation is the absence of biological validation or an external ground truth for CTh. While longitudinal results differed significantly between the two software packages, ultimately, it is unknown which more accurately reflects true CTh changes. Harmonization methods to control for scanner effects were not applicable to our dataset due to the full confounding of scanner and timepoint. However, future work should prioritize scanner consistency or apply statistical harmonization tools when applicable. Furthermore, the present study included only healthy elders. Structural brain imaging analyses are usually used to investigate group differences between patients and controls. In cross-sectional analyses both CAT12 and FreeSurfer were able to detect case-control differences, i.e., thinning related to multiple sclerosis or Alzheimer’s disease [15, 16]. Future studies including careful analysis of cases with mild cognitive impairment, Alzheimer’s disease and other neurological disorders are needed to get more insights on preprocessing-related biases in MRI structural analyses.

## Conclusion

While regional distribution of cross-sectional CTh was similar and correlation high between CAT12 and FreeSurfer processing, longitudinal results differed significantly and showed only weak to moderate correlation. Hence, it is crucial to discuss the preprocessing methods used and to interpret results accordingly. Further technical advances are needed to mitigate scanner effects introduced to CTh measurements.

## Data Availability

Participant data used in this study are not publicly available but stored by the principal investigators of the Geneva Aging Study.

## Abbreviations

APC: Annualized percent change
CAT12: Computational Anatomy Toolbox 12
CI: Confidence interval
CTh: Cortical thickness
FDR: False discovery rate
MPRAGE: Magnetization prepared rapid acquisition gradient echo
MRI: Magnetic resonance imaging
ROI: Region of interest
SD: Standard deviation
T1w: T1–weighted
TR: Repetition time
